# PDA Treatment Strategy and Pulmonary Hemorrhage Risk in Very Low Birth Weight Infants: A Nationwide Cohort Study

**DOI:** 10.3390/children13080991

**Published:** 2026-07-26

**Authors:** Ju Ae Shin, Min Soo Kim, Moon-Yeon Oh

**Affiliations:** Department of Pediatrics, Seoul St. Mary’s Hospital, College of Medicine, The Catholic University of Korea, Seoul 06591, Republic of Korea

**Keywords:** pulmonary hemorrhage, patent ductus arteriosus, very low birth weight, propensity score, neonatal

## Abstract

**Highlights:**

**What are the main findings?**

- In a national cohort of 23,500 VLBW infants, a recorded pre-symptomatic PDA treatment strategy was consistently associated with lower observed pulmonary hemorrhage risk than symptomatic treatment (OR 0.57, 95% CI 0.46–0.72), across multiple sensitivity analyses.
- Prophylactic PDA treatment showed no protective association and carried higher pulmonary hemorrhage risk than pre-symptomatic treatment (OR 1.97, 95% CI 1.31–2.96), likely reflecting selection of high-risk infants.

**What are the implications of the main findings?**

- Prophylactic and pre-symptomatic treatment carry distinct pulmonary hemorrhage risk profiles; “early treatment” should not be treated as a single category.
- These hypothesis-generating findings suggest that risk-based, pre-symptomatic PDA strategies merit prospective evaluation; they do not support universal prophylaxis as a pulmonary hemorrhage prevention measure in VLBW infants.

**Abstract:**

**Background/Objectives:** Pulmonary hemorrhage (PH) is a life-threatening complication of prematurity associated with patent ductus arteriosus (PDA). Whether PDA treatment timing reduces PH risk remains uncertain because treatment strategies reflect underlying illness severity. We examined PH risk across recorded PDA treatment strategy categories in a national very low birth weight (VLBW) cohort. **Methods:** We analyzed Korean Neonatal Network (KNN) registry data (2013–2024) using propensity score inverse probability weighting (IPW) to compare recorded PDA treatment strategies among VLBW infants. Sensitivity analyses addressed treatment-opportunity bias, temporal confounding, and positivity violations. **Results:** Among 23,500 VLBW infants, 1179 (5.0%) developed PH. PH rates were 4.5% (pre-symptomatic), 10.0% (symptomatic treated), 12.3% (symptomatic untreated), 9.9% (prophylactic), and 2.3% (asymptomatic untreated). In IPW analyses, pre-symptomatic treatment consistently showed lower PH risk versus symptomatic treatment (OR 0.57, 95% CI 0.46–0.72) and symptomatic untreated PDA (OR 0.51, 95% CI 0.39–0.66), across sensitivity analyses. Prophylactic treatment showed higher PH risk than pre-symptomatic treatment (OR 1.97, 95% CI 1.31–2.96) and did not differ from symptomatic treatment. Subgroup analyses showed stronger associations in moderately preterm infants, with significant effect modification by gestational age (interaction *p* = 0.011) and birth weight (interaction *p* = 0.007). **Conclusions:** In this national VLBW cohort, infants with a recorded pre-symptomatic PDA treatment strategy had lower observed pulmonary hemorrhage rates compared with those receiving symptomatic treatment, after propensity score adjustment. Prophylactic treatment was not associated with lower PH risk compared with symptomatic treatment but showed higher PH risk than pre-symptomatic treatment. Given the observational design and absence of PH event timing data, causal inferences cannot be drawn. These hypothesis-generating findings support prospective evaluation of risk-based, pre-symptomatic PDA treatment strategies and do not support universal prophylaxis as a pulmonary hemorrhage prevention measure in VLBW infants.

## 1. Introduction

Pulmonary hemorrhage is a severe and often catastrophic complication in preterm infants, particularly among very low birth weight (VLBW) and extremely low birth weight infants. Its pathogenesis is increasingly understood as multifactorial rather than attributable to a single lesion. Immature pulmonary vasculature, respiratory distress syndrome (RDS), surfactant exposure, mechanical ventilation, sepsis, coagulation disturbance, low Apgar scores, and hemodynamic instability may all contribute to pulmonary capillary leak and hemorrhagic pulmonary edema [1,2,3,4,5]. Within this multifactorial framework, patent ductus arteriosus (PDA) remains one of the most biologically plausible and clinically actionable contributors.

The proposed mechanism linking PDA to pulmonary hemorrhage is coherent. After birth, and especially after surfactant therapy, pulmonary vascular resistance may fall abruptly. In the presence of a large left-to-right ductal shunt, pulmonary blood flow can increase, left atrial and pulmonary venous pressures may rise, and the immature pulmonary microvasculature may develop edema that progresses to hemorrhagic edema [1,4,6]. Early observational studies reported that PDA was more common in infants with pulmonary hemorrhage after surfactant treatment [7,8]. More recent reviews and guidelines continue to identify hemodynamically significant PDA (hsPDA) as a recurrent risk factor for pulmonary hemorrhage, while emphasizing that causality remains difficult to prove in observational data [1,4,6,9]. Large national cohort studies, including analyses using the Korean Neonatal Network database, have confirmed symptomatic PDA as an independent risk factor for massive pulmonary hemorrhage [10,11].

The treatment literature is similarly nuanced. Prophylactic indomethacin reduces symptomatic PDA, PDA ligation, and severe intraventricular hemorrhage, and analyses from the Trial of Indomethacin Prophylaxis in Preterms suggested a reduction in early serious pulmonary hemorrhage [12,13]. However, routine prophylactic PDA treatment has not consistently improved mortality or long-term neurodevelopmental outcomes [12,14]. Prophylactic ibuprofen decreases PDA-related short-term outcomes but has not established broader outcome benefits [15]. Acetaminophen (paracetamol), which inhibits prostaglandin synthesis at the peroxidase site rather than the cyclooxygenase site targeted by indomethacin and ibuprofen, has emerged as an alternative PDA pharmacotherapy, particularly in settings where cyclooxygenase inhibitors are contraindicated, although evidence on efficacy and safety remains heterogeneous across doses, routes, and gestational age groups [16,17,18,19]. In terms of closure efficacy, a network meta-analysis of pharmacologic PDA treatments found that oral ibuprofen and acetaminophen achieve ductal closure rates at least comparable to intravenous indomethacin, with a high dose of oral ibuprofen associated with the highest odds of closure, though comparative effects on major clinical outcomes such as mortality and bronchopulmonary dysplasia remain uncertain [17]. Beyond closure efficacy, more recent randomized trials of early targeted treatment, including PDA-TOLERATE, BeNeDuctus, and Baby-OSCAR, have further complicated the field by showing that routine early pharmacologic closure of PDA does not necessarily translate into improved composite outcomes such as death or bronchopulmonary dysplasia [20,21,22]. A 2025 meta-analysis of 10 randomized trials found that active closure of hemodynamically significant PDA during the first 2 weeks of life was associated with higher mortality compared with expectant management, further discouraging non-selective closure [23]. Thus, contemporary PDA management has moved away from universal closure and toward risk-based strategies using clinical instability, respiratory status, and echocardiographic evidence of hemodynamic significance [6,9,24,25,26].

A major limitation of prior observational studies is that PDA treatment is often analyzed as a binary exposure, despite representing heterogeneous clinical states. Prophylactic treatment, pre-symptomatic treatment, symptomatic treatment, symptomatic untreated PDA, and asymptomatic untreated PDA likely reflect different combinations of ductal physiology, clinician perception of risk, contraindications to therapy, and timing of clinical deterioration. Combining these categories may obscure clinically meaningful patterns. In particular, prophylactic treatment may be preferentially used in infants already considered high risk, whereas pre-symptomatic treatment may represent early intervention before overt cardiorespiratory decompensation. Conversely, symptomatic untreated PDA may include infants who were too unstable, had contraindications, or died before treatment opportunity.

Using a national VLBW cohort, we examined PH risk across recorded PDA treatment strategies. Our aim was descriptive and hypothesis-generating—to characterize how PH risk is distributed across these clinically distinct management phenotypes, and in particular to test whether prophylactic and pre-symptomatic treatment, which prior binary analyses have combined as “early treatment,” carry distinct PH risk profiles—rather than to estimate the causal effect of any single treatment on PH. We hypothesized that a recorded pre-symptomatic PDA treatment strategy would be associated with lower PH risk compared with symptomatic treatment or symptomatic untreated PDA, whereas prophylactic treatment would not necessarily show a protective association because of confounding by indication.

## 2. Materials and Methods

### 2.1. Study Design and Data Source

This was a retrospective cohort study using Korean Neonatal Network (KNN) registry data from 2013 to 2024. The KNN is a national multicenter quality improvement registry collecting clinical data on very low birth weight infants from participating neonatal intensive care units across Korea [27]. Variable definitions followed established KNN coding specifications.

### 2.2. Study Population

The main cohort included infants without major congenital anomalies and with a documented PDA treatment strategy (prophylactic, pre-symptomatic, symptomatic treatment, symptomatic untreated, or asymptomatic untreated). Infants with unknown or unrecorded PDA treatment strategy were excluded from the main analysis.

### 2.3. Exposure: PDA Treatment Strategy

PDA treatment strategy was classified into five categories based on the KNN recorded treatment strategy variable. **Prophylactic treatment** was defined as pharmacologic treatment initiated before any clinical or echocardiographic evaluation of PDA—administered as institutional policy without diagnostic criteria. **Pre-symptomatic treatment** was defined as treatment initiated after echocardiographic or biomarker confirmation of PDA presence in the absence of overt cardiorespiratory symptoms. **Symptomatic treatment** was defined as treatment initiated after overt clinical signs of hemodynamically significant PDA; in the KNN, this required at least two of the following five criteria together with color-flow Doppler echocardiographic confirmation of large left-to-right ductal flow: (1) systolic or continuous cardiac murmur; (2) bounding peripheral pulses or hyperactive precordial pulsation; (3) hypotension unresponsive to fluid or inotropic therapy; (4) respiratory deterioration; or (5) chest radiograph evidence of pulmonary congestion or cardiomegaly (cardiothoracic ratio > 60%). Echocardiographic assessment of PDA significance in the KNN incorporated shunt direction and ductal hemodynamic significance as primary diagnostic elements, consistent with established criteria for hemodynamically significant PDA [9,24,25]. **Symptomatic untreated PDA** was defined as meeting the above clinical criteria without pharmacologic treatment (supportive care only). **Asymptomatic untreated PDA** was defined as absence of the above criteria with no pharmacologic treatment.

The KNN records surgical ductal ligation as a separate variable. Surgical ligation was not used as an exclusion criterion; ligation rates by treatment strategy are described in **Section 3**.

The primary comparison was pre-symptomatic treatment versus symptomatic treatment. Key secondary comparisons were pre-symptomatic treatment versus symptomatic untreated PDA, prophylactic treatment versus pre-symptomatic treatment, and prophylactic treatment versus symptomatic treatment.

### 2.4. Outcome

The primary outcome was pulmonary hemorrhage, defined using the KNN clinical variable for massive pulmonary hemorrhage (recorded as present when clinically confirmed pulmonary bleeding required intervention). Secondary descriptive outcomes included in-hospital mortality, length of stay, and postnatal day of first PDA drug administration. Postnatal day of first PDA drug administration was calculated from the date and time of first drug dose and date of birth, both recorded in the KNN, and summarized descriptively by treatment strategy as a secondary characterization of treatment timing.

### 2.5. Statistical Analysis

Propensity score covariates were selected a priori: gestational age, birth weight, sex, multiple birth, antenatal steroid exposure, Cesarean delivery, 1 min and 5 min Apgar scores, resuscitation severity, respiratory distress syndrome, surfactant treatment, and hypotension. The last three were included as early clinical severity markers that may also lie on the causal pathway; a sensitivity analysis using perinatal baseline covariates only (excluding these three) was performed alongside the main model.

PH rates, mortality rates, and surgical ligation rates were first summarized by strategy using descriptive statistics. For pairwise comparisons, propensity scores were estimated by logistic regression and stabilized inverse probability weights were applied with 1st–99th percentile trimming. Covariate balance was assessed by standardized mean differences (SMDs), with absolute SMD < 0.1 considered acceptable. Pairwise IPW models estimated odds ratios (ORs), 95% confidence intervals (CIs), and weighted absolute risk differences for PH. No formal correction for multiple comparisons was applied; the pre-symptomatic versus symptomatic treatment comparison was pre-specified as primary, and remaining pairwise comparisons are considered secondary or exploratory. All pairwise IPW analyses used complete cases with respect to propensity score covariates; infants with missing covariate values were excluded from each pairwise comparison (*n* = 49 for the primary pre-symptomatic vs. symptomatic treated comparison, yielding *n* = 7049). Drug-type analyses excluded 654 infants with an unrecorded drug type.

Sensitivity analyses addressed: (1) treatment-opportunity bias, by excluding infants who died by day 3 or day 7; (2) temporal practice changes, by adding birth year to the PS model; (3) possible overadjustment, using the perinatal baseline-only PS model; and (4) potential positivity violations, using overlap (ATO) weights—in which each observation is weighted by P(A = 0|X) for the exposed group and P(A = 1|X) for the reference group—and PS-restricted IPW limited to propensity scores 0.10–0.90. Propensity score distributions for each pairwise comparison are shown in Figure S2.

Exploratory subgroup analyses used pre-symptomatic treatment versus symptomatic treatment, consistent with the primary pairwise comparison. Covariate-adjusted logistic regression with interaction terms was used to assess effect modification by gestational age, birth weight, surfactant use, respiratory distress syndrome, and hypotension. Drug-type analyses (indomethacin, ibuprofen, and acetaminophen) were restricted to pharmacologically treated infants and were considered exploratory given small non-ibuprofen groups.

Analyses were performed using Python (version 3.12.6; Python Software Foundation, Wilmington, DE, USA) with the pandas, NumPy, statsmodels, SciPy, Matplotlib, and seaborn libraries.

## 3. Results

### 3.1. Cohort and PDA Treatment Strategy Distribution

**Figure 1:**
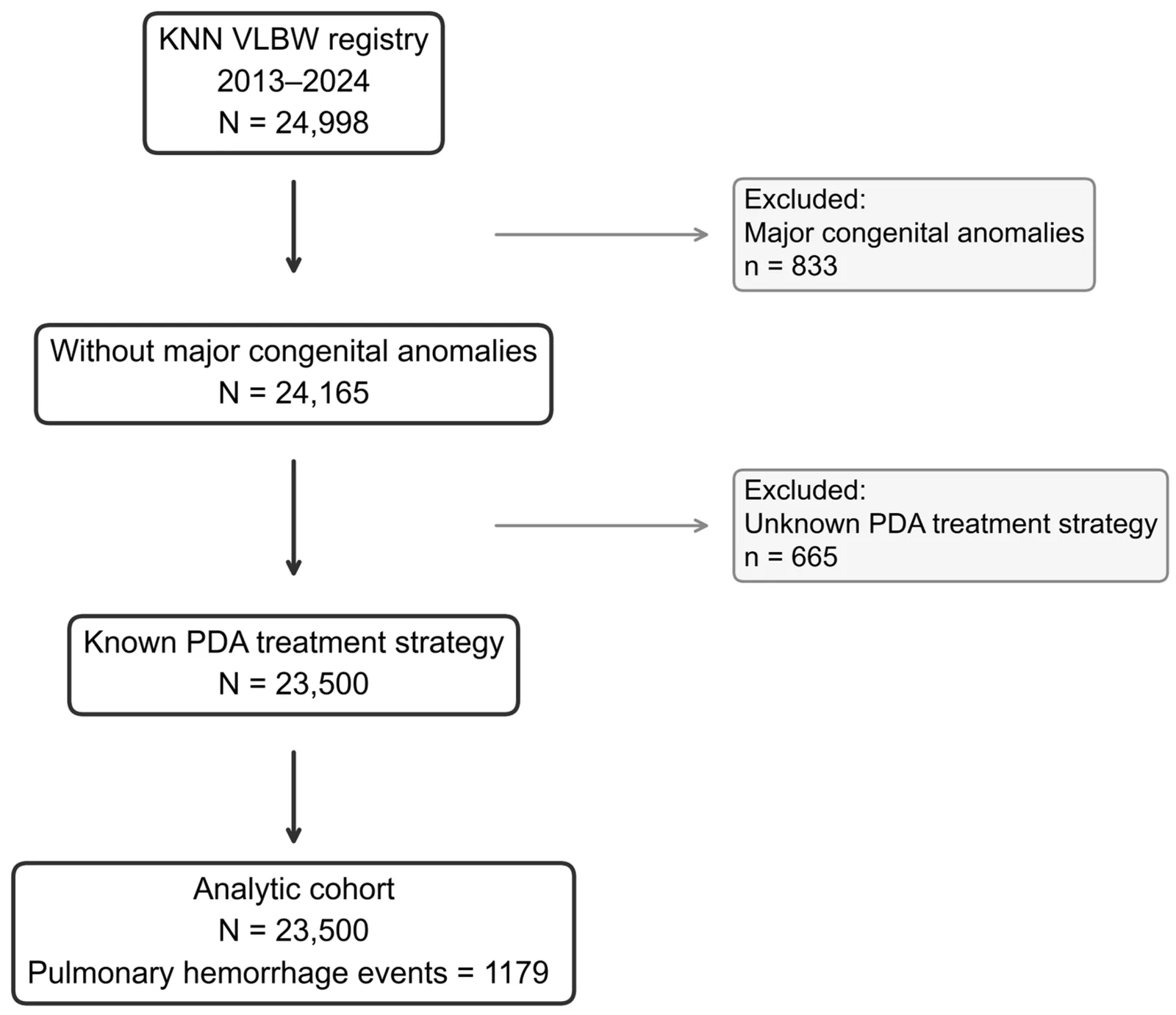
Study flow diagram. Flow of very low birth weight (VLBW) infants from the Korean Neonatal Network (KNN) registry (2013–2024) into the main analytic cohort, after sequential exclusion of infants with major congenital anomalies and those with unknown recorded patent ductus arteriosus (PDA) treatment strategy. The number of pulmonary hemorrhage events in the analytic cohort is shown. KNN, Korean Neonatal Network; PDA, patent ductus arteriosus; VLBW, very low birth weight.

The study flow is shown in Figure 1. The main cohort included 23,500 VLBW infants without major congenital anomalies and with recorded PDA strategy categories. PH occurred in 1179 infants. PDA treatment strategies were distributed as follows: prophylactic treatment in 414 infants, pre-symptomatic treatment in 1775, symptomatic treatment in 5323, symptomatic untreated PDA in 1565, and asymptomatic untreated PDA in 14,423. Baseline characteristics differed across PDA strategy groups (Table 1). Infants with symptomatic untreated PDA were the most immature by median gestational age and birth weight, whereas asymptomatic untreated infants had the highest median gestational age and birth weight.

### 3.2. Pulmonary Hemorrhage and Mortality by PDA Strategy

**Figure 2:**
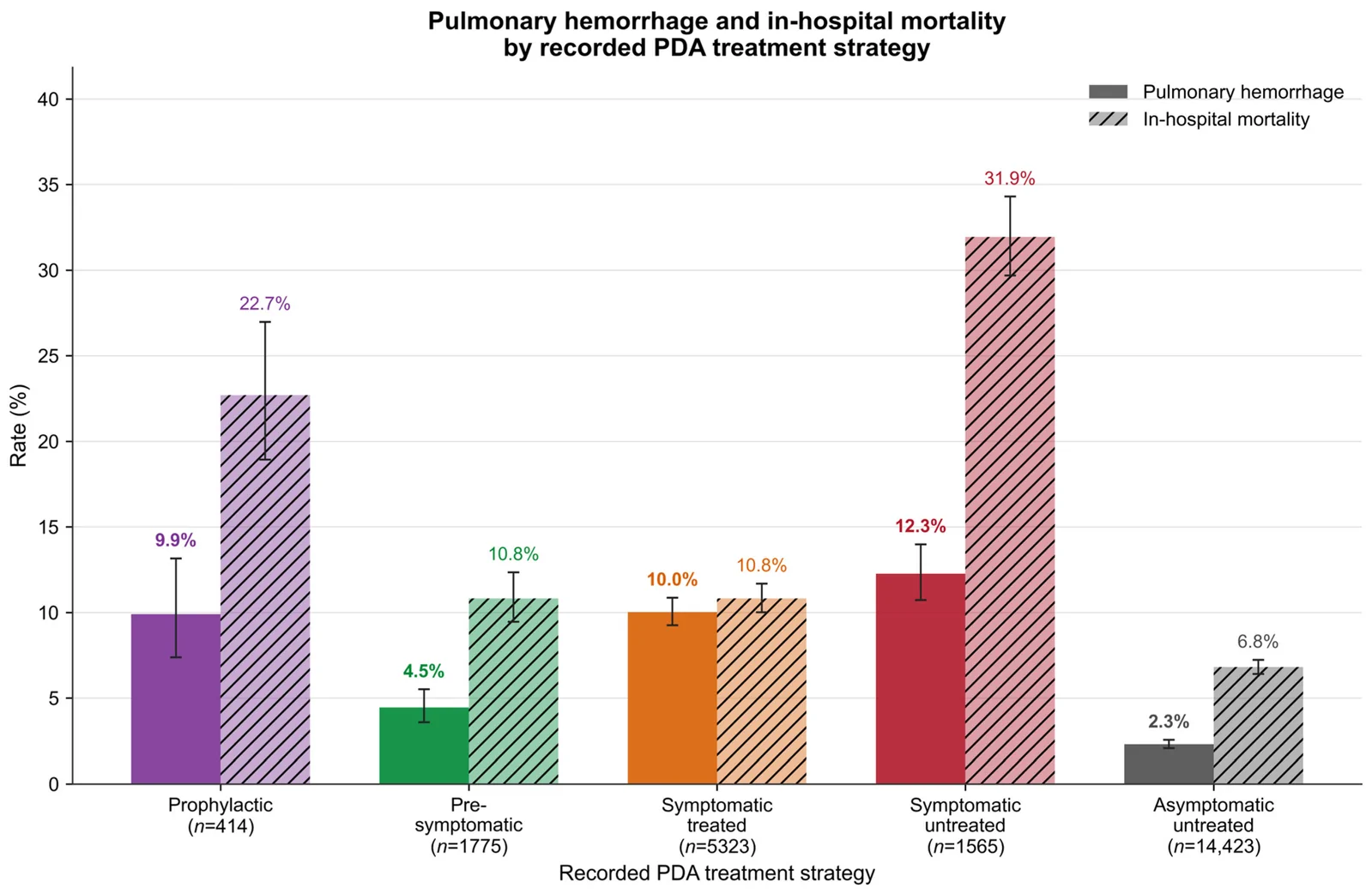
Pulmonary hemorrhage and in-hospital mortality by recorded PDA treatment strategy. Observed rates of pulmonary hemorrhage (solid bars) and in-hospital mortality (hatched bars) across five recorded PDA treatment strategy categories: prophylactic, pre-symptomatic, symptomatic treated, symptomatic untreated, and asymptomatic untreated. Asymptomatic untreated infants represent a low-risk reference phenotype rather than a direct untreated comparator. Error bars indicate Wilson 95% confidence intervals. PDA, patent ductus arteriosus; PH, pulmonary hemorrhage.

PH rates varied markedly by PDA treatment strategy (Table 2 and Figure 2). PH was lowest in asymptomatic untreated infants (2.31%) and pre-symptomatic treatment infants (4.45%). In contrast, PH occurred in 10.03% of symptomatic treatment infants and 12.27% of symptomatic untreated infants. Prophylactic treatment infants had a PH rate of 9.90%, similar to symptomatic treatment infants.

Mortality also differed by strategy. Symptomatic untreated infants had high mortality (31.95%), supporting the interpretation that this group may represent a high-risk phenotype with treatment opportunity bias. Early death rates by PDA strategy are detailed in Table S1. Prophylactic treatment infants also had higher mortality (22.71%) than pre-symptomatic or symptomatic treatment infants (both 10.82%).

Surgical ductal ligation was performed in 2092 infants (8.9% of the main cohort). Ligation rates differed substantially by treatment strategy: 33.4% in symptomatic treatment, 14.9% in pre-symptomatic treatment, and 12.1% in prophylactic treatment infants; no ligation was recorded among symptomatic untreated or asymptomatic untreated infants. Among infants who underwent ligation, PH rates were higher than in those who did not within each strategy group: 14.3% versus 7.9% in symptomatic treatment, and 11.7% versus 3.2% in pre-symptomatic treatment.

### 3.3. Pairwise IPW Analyses

After IPW, covariate balance was excellent across all key pairwise comparisons. Maximum absolute SMD after weighting ranged from 0.009 to 0.055, and no comparison had any covariate with absolute SMD > 0.1 (Figure S1).

**Figure 3:**
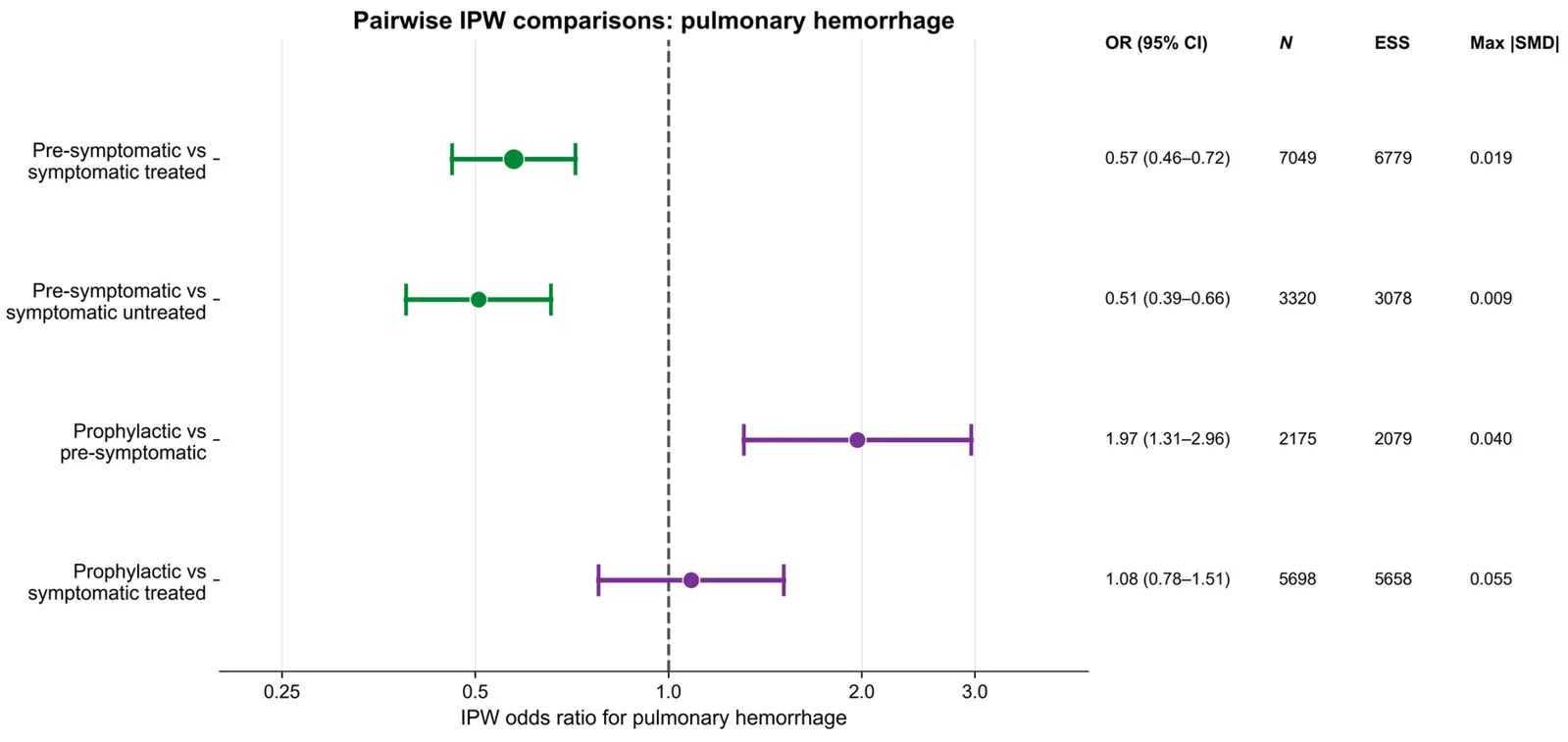
***Pairwise inverse probability weighting (IPW) forest plot.*** IPW odds ratios for pulmonary hemorrhage in four key pairwise comparisons of recorded PDA treatment strategy. IPW weights were stabilized and trimmed at the 1st–99th percentiles. All comparisons achieved excellent post-weighting covariate balance (maximum |SMD| < 0.1 for all covariates). Marker and interval colors indicate the index treatment strategy of each comparison (green, pre-symptomatic; purple, prophylactic). CI, confidence interval; ESS, effective sample size; IPW, inverse probability weighting; OR, odds ratio; PDA, patent ductus arteriosus; PH, pulmonary hemorrhage; SMD, standardized mean difference.

A recorded pre-symptomatic treatment strategy was associated with lower PH risk compared with symptomatic treatment (IPW OR 0.57, 95% CI 0.46–0.72; absolute risk difference [RD] −3.8 percentage points [pp], 95% CI −5.3 to −2.4) and symptomatic untreated PDA (IPW OR 0.51, 95% CI 0.39–0.66; RD −4.9 pp, 95% CI −6.7 to −3.1). Prophylactic treatment was associated with higher PH risk than pre-symptomatic treatment (IPW OR 1.97, 95% CI 1.31–2.96; RD +4.1 pp, 95% CI 1.2 to 7.3) but had similar PH risk compared with symptomatic treatment (IPW OR 1.08, 95% CI 0.78–1.51; RD +0.8 pp, 95% CI −2.4 to 4.3). Pairwise IPW results are summarized in Table 3 and Figure 3.

Positivity sensitivity analyses confirmed the robustness of the primary pre-symptomatic comparisons. For pre-symptomatic versus symptomatic treatment, 97.8% of infants were within the PS overlap/restriction region (0.10–0.90); overlap (ATO) weights yielded OR 0.55 (95% CI 0.40–0.76) and PS-restricted IPW yielded OR 0.58 (95% CI 0.46–0.72, *n* = 6897). For pre-symptomatic versus symptomatic untreated PDA, 100% were within the overlap region with consistent estimates across methods (overlap: OR 0.51, 95% CI 0.35–0.74; PS-restricted: OR 0.51, 95% CI 0.39–0.66). In contrast, only 16.3% of infants were within the overlap region for the prophylactic versus symptomatic treatment comparison (Figure S2); the PS-restricted estimate (OR 0.97, 95% CI 0.45–2.12, *n* = 926) indicated substantial extrapolation in the primary IPW and should be interpreted cautiously. For prophylactic versus pre-symptomatic treatment, 88.7% were within the overlap region; the PS-restricted estimate (OR 1.76, 95% CI 1.16–2.67, *n* = 1930) was consistent in direction with the primary estimate (Table S2).

Sensitivity analyses supported the robustness of the primary findings. Birth-year adjustment did not change the estimates for pre-symptomatic versus symptomatic treatment (OR 0.57, 95% CI 0.46–0.72) or symptomatic untreated PDA (OR 0.51, 95% CI 0.39–0.66). After excluding infants who died by day 3, the corresponding ORs were 0.55 (95% CI 0.43–0.69) and 0.55 (95% CI 0.41–0.72); after excluding those who died by day 7, ORs were 0.53 (95% CI 0.42–0.68) and 0.59 (95% CI 0.43–0.79) (Table S3). In the baseline-only PS sensitivity analysis, pre-symptomatic treatment remained associated with lower PH risk versus symptomatic treatment (OR 0.49, 95% CI 0.39–0.62) and symptomatic untreated PDA (OR 0.44, 95% CI 0.34–0.58); prophylactic treatment remained associated with higher PH risk than pre-symptomatic treatment (OR 2.04, 95% CI 1.36–3.05) and was not different from symptomatic treatment (OR 0.98, 95% CI 0.70–1.38; Table S4).

### 3.4. PDA Drug Administration Timing

Among pharmacologically treated infants with a recorded date of first PDA drug administration (*n* = 408 prophylactic, 1751 pre-symptomatic, and 4776 symptomatic; infants whose administration date was not recorded were excluded from this timing analysis), the postnatal day of first PDA drug administration differed significantly across strategy groups (Kruskal–Wallis *p* < 0.001). The median day of first drug administration was 1 day (IQR 0–1) for prophylactic, 2 days (IQR 1–4) for pre-symptomatic, and 5 days (IQR 3–10) for symptomatic treatment. Among pre-symptomatic treated infants, 70.3% received their first PDA drug by postnatal day 3, and 88.7% by postnatal day 7. Among symptomatic treated infants, 36.8% received their first drug by day 3 and 64.9% by day 7. These timing differences are consistent with the clinical intent of the recorded strategy categories and confirm that pre-symptomatic treatment was initiated substantially earlier in the postnatal course than symptomatic treatment. The distribution of drug administration timing by strategy is shown in Figure S4.

### 3.5. Subgroup Analyses

Exploratory subgroup analyses examined pre-symptomatic treatment (*n* = 1775) versus symptomatic treatment (*n* = 5323) using covariate-adjusted logistic regression. The association between pre-symptomatic treatment strategy and lower PH risk was more prominent in moderately immature infants than in the most immature infants. Among infants 28–31 weeks’ gestation, pre-symptomatic treatment was associated with substantially lower PH risk (OR 0.34, 95% CI 0.20–0.58), whereas the association was smaller among infants < 28 weeks (OR 0.61, 95% CI 0.46–0.82). Similarly, the association was not significant among infants < 750 g (OR 0.73, 95% CI 0.50–1.06), but was present among infants 750–999 g (OR 0.50, 95% CI 0.33–0.78) and 1000–1499 g (OR 0.29, 95% CI 0.16–0.52).

**Figure 4:**
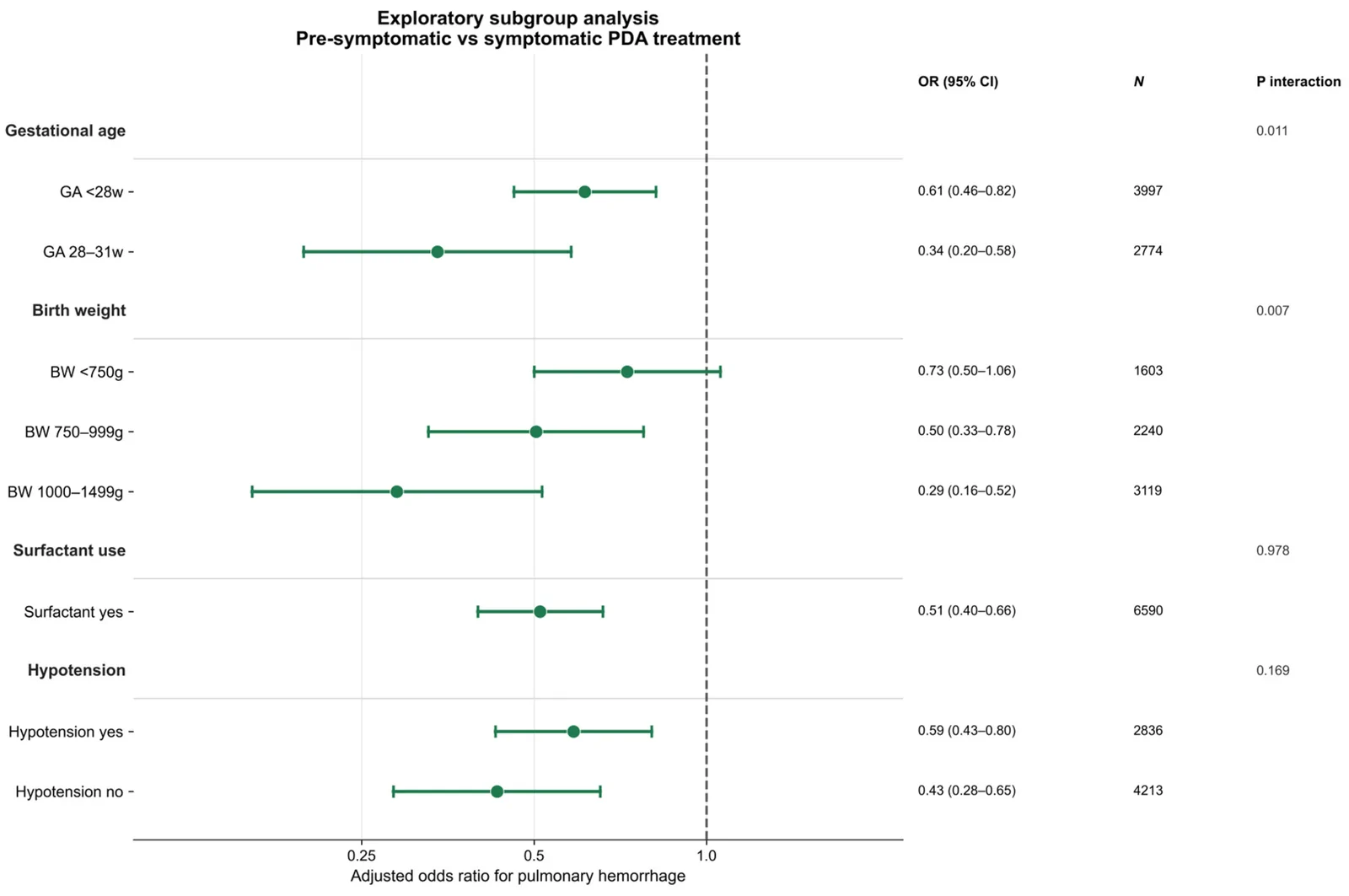
***Exploratory subgroup forest plot.*** Covariate-adjusted odds ratios for pulmonary hemorrhage, comparing pre-symptomatic versus symptomatic PDA treatment within subgroups defined by gestational age, birth weight, surfactant use, and hypotension status. This comparison is consistent with the primary pairwise IPW analysis (Figure 3). Interaction *p*-values test binary effect modification within each domain. BW, birth weight; CI, confidence interval; GA, gestational age; IPW, inverse probability weighting; OR, odds ratio; PDA, patent ductus arteriosus; PH, pulmonary hemorrhage.

Interaction tests supported effect modification by gestational age (interaction *p* = 0.011) and birth weight (interaction *p* = 0.007). Subgroup odds ratios are summarized in Table 4 and Figure 4. Interactions by surfactant exposure (interaction *p* = 0.978), respiratory distress syndrome (interaction *p* = 0.528), and hypotension status (interaction *p* = 0.169) were not significant.

### 3.6. PDA Drug Type

Among infants receiving PDA drug therapy, ibuprofen was the dominant medication. PH occurred in eight of 78 indomethacin-treated infants (10.26%), 504 of 6,645 ibuprofen-treated infants (7.58%), and 25 of 135 acetaminophen-treated infants (18.52%; Figure S3). In exploratory adjusted analyses, acetaminophen treatment was associated with higher PH risk compared with ibuprofen (adjusted OR 2.08, 95% CI 1.30–3.32). Indomethacin was not significantly different from ibuprofen (adjusted OR 1.47, 95% CI 0.68–3.17). Because acetaminophen and indomethacin groups were small and likely confounded by treatment selection, these findings should be interpreted as hypothesis-generating.

Detailed drug-type counts and regression models are provided in Tables S5 and S6.

## 4. Discussion

### 4.1. Principal Findings

In this national VLBW cohort, PH risk differed substantially across recorded PDA treatment strategies. A recorded pre-symptomatic PDA treatment strategy was associated with lower observed PH risk compared with symptomatic treatment and symptomatic untreated PDA, even after propensity score weighting with excellent covariate balance. These associations persisted after birth-year adjustment, after excluding infants who died by day 3 or day 7, and when the PS model was restricted to perinatal baseline covariates. In contrast, prophylactic treatment did not show a protective association; PH risk in prophylactically treated infants was similar to that in symptomatic treatment infants and higher than that in pre-symptomatic treatment infants.

These findings suggest that PDA treatment timing and clinical context are critical. The category of “early treatment” is not homogeneous. A recorded pre-symptomatic strategy may capture a clinically meaningful window in which PDA treatment is intended before overt hemodynamic or respiratory decompensation, whereas prophylactic treatment may identify infants selected for treatment because clinicians already considered them extremely high risk. Because PH event dates were unavailable, this interpretation should be regarded as clinical inference from recorded strategy categories rather than confirmation that treatment preceded PH in every infant. Although median postnatal day of first PDA drug was substantially earlier in the pre-symptomatic group (day 2, IQR 1–4) than the symptomatic group (day 5, IQR 3–10), individual-level temporal precedence between treatment initiation and PH occurrence cannot be established from these registry data, and a causal interpretation is not supported.

Surgical ligation rates differed substantially between strategy groups: 33.4% of symptomatic treatment infants underwent ligation compared with 14.9% of pre-symptomatic treatment infants. Within each group, ligation was associated with higher PH rates (14.3% versus 7.9% in symptomatic treatment; 11.7% versus 3.2% in pre-symptomatic treatment). Because ligation was not included as a PS model covariate—its role as a downstream mediator or confounder is difficult to separate—the between-group difference in ligation rates may partly account for the observed PH differences, and this should be considered when interpreting the primary IPW estimates.

An additional finding deserving attention is the high case fatality among PH-positive infants in the pharmacologically treated groups. This pattern suggests that PH among infants recorded as receiving prophylactic or pre-symptomatic treatment may represent a particularly severe phenotype arising in infants with profound vascular immaturity, coagulopathy, or multi-organ instability that PDA treatment alone cannot modify. This interpretation reinforces the need to avoid over-attributing the lower overall PH rate in pre-symptomatic treatment infants to treatment efficacy alone; unmeasured severity differences between groups likely contribute to the observed association.

### 4.2. Comparison with Previous Literature

Our findings are consistent with earlier reports linking PDA with PH in preterm infants. Garland and colleagues described a higher prevalence of clinically detectable PDA among infants with pulmonary hemorrhage after rescue surfactant therapy [7]. Autopsy-based work after synthetic surfactant similarly found PDA, intraventricular hemorrhage, and pneumothorax to be more frequent among infants who developed PH [8]. These studies helped establish the clinical intuition that PDA-related pulmonary overcirculation can contribute to PH, particularly in surfactant-treated infants.

The randomized trial literature provides a more complicated backdrop. Prophylactic indomethacin has strong short-term physiologic effects and reduces symptomatic PDA and severe intraventricular hemorrhage, but it has not demonstrated clear survival or long-term neurodevelopmental benefit [12]. TIPP secondary analyses suggested that indomethacin prophylaxis reduced early serious pulmonary hemorrhage and that this benefit was partly related to PDA reduction [13]. A more recent cohort study also reported that prophylactic indomethacin was associated with a lower incidence of serious pulmonary hemorrhage in infants born before 28 weeks’ gestation [28]. In contrast, trials of later or targeted treatment have generally not demonstrated consistent improvements in major composite outcomes. PDA-TOLERATE did not establish clear clinical benefit of routine treatment of moderate-to-large PDA at approximately 1 week of age [20]. BeNeDuctus found expectant management to be noninferior to early ibuprofen for a composite of necrotizing enterocolitis, bronchopulmonary dysplasia, or death among extremely preterm infants with echocardiographically confirmed PDA [21]. Baby-OSCAR similarly found that selective early ibuprofen treatment of a large PDA did not reduce death or moderate/severe bronchopulmonary dysplasia [22]. These findings support the modern view that PDA closure itself is not a universally beneficial endpoint. Notably, a 2025 Cochrane systematic review of 19 RCTs comparing early versus expectant management of hemodynamically significant PDA in preterm infants found that pulmonary hemorrhage was not included among the pre-specified primary or secondary outcomes in either comparison [29], consistent with the absence of sufficient PH data from individual trials to enable meta-analysis of this specific complication.

Our study adds an important distinction that is not easily addressed in randomized trials or binary observational analyses: prophylactic treatment and pre-symptomatic treatment should not be combined uncritically. A prior KNN cohort study by Shin et al. found lower neonatal mortality in actively treated PDA groups compared with conservative management, but did not separately evaluate prophylactic and pre-symptomatic categories [30]. In our cohort, the recorded pre-symptomatic treatment strategy showed the strongest association with lower PH risk, whereas prophylactic treatment appeared to reflect a high-risk treatment-selection phenotype. This may explain why broad “early treatment” categories often produce mixed results. The clinically relevant question may not be whether all PDAs should be closed early, but whether selected infants with evolving ductal significance can be treated before they transition to overt pulmonary overcirculation and symptomatic instability.

### 4.3. Biological Plausibility

The association between symptomatic PDA and PH is biologically plausible. After surfactant therapy, pulmonary vascular resistance may fall rapidly, increasing left-to-right ductal shunting. This can increase pulmonary blood flow, left atrial pressure, and pulmonary venous pressure, leading to pulmonary edema and hemorrhagic edema [1,4,6]. Echocardiographic assessment of hsPDA typically considers more than ductal diameter alone, incorporating shunt direction, left heart volume loading, systemic diastolic flow effects, and clinical respiratory status [9,24,25]. A pre-symptomatic treatment strategy may reduce the likelihood that infants progress to overt pulmonary overcirculation and symptomatic PDA. However, this interpretation remains cautious because event timing for PH and echocardiographic severity markers were unavailable in the present dataset. Future prospective studies that couple standardized echocardiographic phenotyping—including ductal diameter, left atrial-to-aortic root ratio, and transductal flow patterns—with prospectively recorded pulmonary hemorrhage timing will be needed to define the echocardiographic thresholds that best identify infants likely to benefit from pre-symptomatic treatment.

### 4.4. Subgroup Findings

Exploratory subgroup analyses of pre-symptomatic versus symptomatic treatment showed significant effect modification by gestational age (interaction *p* = 0.011) and birth weight (interaction *p* = 0.007). The association with lower PH risk was weaker and narrowed toward the null among the most immature infants (<28 weeks, OR 0.61) and smallest infants (<750 g, OR 0.73, not significant), whereas it was stronger among infants of 28–31 weeks (OR 0.34) or 1000–1499 g (OR 0.29). This pattern is clinically plausible: in the most immature infants, PH may be driven by multiple severe illness pathways—extreme vascular immaturity, severe RDS, mechanical ventilation, coagulation abnormalities, and systemic instability—that PDA treatment alone cannot modify. In moderately immature infants, PDA-related pulmonary overcirculation may account for a larger modifiable fraction of PH risk. Interactions by hypotension status, surfactant use, and RDS were not significant. These subgroup findings should be tested in datasets with echocardiographic PDA severity and PH event timing.

### 4.5. Drug-Type Findings

The drug-type analysis suggested higher PH rates among acetaminophen-treated infants compared with ibuprofen-treated infants. This should not be interpreted as evidence of acetaminophen toxicity. Trials and systematic reviews suggest that acetaminophen can close PDA, although evidence differs by dose, route, comparator, and infant maturity [16,17,18,19]. In routine practice, acetaminophen may be used as rescue therapy or in infants with contraindications to cyclooxygenase inhibitors. In this cohort, acetaminophen-treated infants had a lower median gestational age (26 weeks) and lower median birth weight (800 g) than ibuprofen-treated infants (27 weeks; 980 g), indicating that differences in baseline maturity and illness severity contribute substantially to the higher observed PH rate in the acetaminophen group. The small sizes of the indomethacin and acetaminophen groups further limit inference. Drug-specific findings should therefore be considered hypothesis-generating and secondary to the strategy-based findings.

### 4.6. Strengths

Strengths of this study include the use of one of the largest national VLBW registry cohorts to examine pulmonary hemorrhage by PDA treatment strategy (*N* = 23,500 infants; 2013–2024), a five-category treatment strategy classification that captures clinically distinct management approaches unavailable in prior binary analyses, and propensity score inverse probability weighting with strong post-weighting covariate balance (maximum |SMD| < 0.1 for all pairwise comparisons). Multiple pre-specified sensitivity analyses—including early death exclusion at postnatal day 3 and day 7, birth-year adjustment, baseline-only propensity score modeling, and positivity-restricted analyses using overlap (ATO) weights and PS-restricted IPW—consistently supported the primary association. The availability of drug administration dates in the KNN further enabled characterization of treatment timing by strategy group, supporting the construct validity of the exposure classification. The 12-year study period (2013–2024) spanning substantial evolution in neonatal PDA management provides a representative contemporary sample and adequate statistical power for this low-prevalence outcome.

### 4.7. Limitations

This study has several important limitations. First and most importantly, PH event dates were not recorded in the KNN registry, precluding confirmation that treatment preceded the PH event at the individual patient level. Although median postnatal day of first PDA drug was 2 days (IQR 1–4) for pre-symptomatic and 5 days (IQR 3–10) for symptomatic treatment—consistent with the temporal intent of the recorded strategy categories—this group-level difference does not establish that treatment preceded PH for individual infants, and a causal interpretation cannot be supported. Because PH is also a competing-risk-sensitive outcome, early death may further distort observed associations if infants die before treatment opportunity or before PH can be diagnosed.

Second, echocardiographic PDA severity measures—including ductal diameter, left atrial-to-aortic root ratio, diastolic flow reversal, and other indices of hemodynamic significance—were unavailable as discrete registry variables and could not be incorporated into the propensity score model. Additional clinical severity indicators, including ventilator settings and fractional inspired oxygen requirements, were also unavailable. Residual confounding by true ductal hemodynamic burden and unmeasured clinical severity therefore cannot be excluded.

Third, the KNN symptomatic classification requires respiratory deterioration as one of five diagnostic criteria; acute PH could itself precipitate respiratory deterioration, introducing the possibility of reverse causality in the symptomatic groups that echocardiographic confirmation of large ductal flow only partially mitigates. This bidirectional relationship between PH and respiratory decompensation represents an important source of potential misclassification that cannot be resolved in the present dataset.

Fourth, PDA treatment strategy is inherently subject to confounding by indication; treatment decisions reflect physician assessment of risk, clinical instability, and institutional practice patterns that cannot be fully captured by measured covariates. Although IPW achieved excellent covariate balance and multiple sensitivity analyses were consistent, unmeasured severity likely contributes to the observed associations. Selection into each recorded category, moreover, is non-random and unlikely to operate in a single direction. Classification as symptomatic treated, for example, is conditional on an infant surviving without treatment until overt symptoms emerge and then being stable enough to receive therapy—a survival- and stability-based selection that may enrich this group for later-onset or less fulminant ductal presentations—whereas pre-symptomatic and prophylactic treatment are driven by clinician anticipation of risk before decompensation. These opposing and unmeasured selection pressures cannot be separated with the available data and further reinforce that the between-group differences are hypothesis-generating rather than causal. Propensity score overlap was severely limited for the prophylactic versus symptomatic treatment comparison (16.3% within the PS overlap/restriction region, 0.10–0.90), so that the primary IPW estimate required substantial extrapolation and should be interpreted cautiously; the PS-restricted estimate was imprecise (OR 0.97, 95% CI 0.45–2.12, *n* = 926). The main PS model was also adjusted for RDS, surfactant, and hypotension, which may partly lie on the causal pathway; the baseline-only sensitivity analysis (Table S4) should be considered alongside the main model.

Fifth, center identifiers were unavailable, preventing center-level adjustment; prophylactic treatment may partly reflect institutional policy. Surgical ligation rates also differed substantially across groups (33.4% in symptomatic treatment versus 14.9% in pre-symptomatic treatment) and were not formally decomposed, as ligation likely represents a downstream treatment step rather than a confounder. Sixth, drug-type analyses were exploratory and strongly confounded by treatment selection.

The KNN recorded PDA treatment strategy represents clinician-documented intent rather than a direct physiologic measure of hemodynamic significance. The “symptomatic untreated” category is particularly complex and may include infants with contraindications, rapidly fatal illness, or treatment opportunity loss, and should not be equated with elective conservative management.

## 5. Conclusions

In this national VLBW cohort, infants with a recorded pre-symptomatic PDA treatment strategy had lower observed pulmonary hemorrhage rates compared with those receiving symptomatic treatment, after propensity score adjustment and across multiple sensitivity analyses. Prophylactic PDA treatment was not associated with lower pulmonary hemorrhage risk compared with symptomatic treatment, showed higher pulmonary hemorrhage risk than pre-symptomatic treatment (OR 1.97, 95% CI 1.31–2.96), and likely identified a clinically selected high-risk subgroup. These findings are hypothesis-generating; because pulmonary hemorrhage event timing was unavailable and echocardiographic severity measures were not captured in the registry, causal inference is not supported. Prospective evaluation of risk-based, pre-symptomatic PDA treatment strategies is warranted, while universal prophylaxis is not supported as a pulmonary hemorrhage prevention measure in VLBW infants.

## Figures and Tables

**Table 1:** Baseline characteristics of VLBW infants by recorded PDA treatment strategy.

Characteristic	Prophylactic (*n* = 414)	Pre-Symptomatic (*n* = 1775)	Symptomatic Treated (*n* = 5323)	Symptomatic Untreated (*n* = 1565)	Asymptomatic Untreated (*n* = 14,423)	*p* Value
Gestational age, weeks	27.4 [25.4, 29.1]	28.0 [26.1, 29.6]	27.3 [25.7, 29.0]	26.6 [24.7, 28.9]	30.1 [28.4, 31.7]	<0.001
Birth weight, g	985 [750, 1200]	1020 [800, 1242]	940 [760, 1160]	840 [630, 1120]	1267 [1030, 1420]	<0.001
Male sex, *n* (%)	221 (53.4%)	835 (47.0%)	2613 (49.1%)	806 (51.5%)	7434 (51.5%)	<0.001
Multiple birth, *n* (%)	131 (31.6%)	673 (37.9%)	2061 (38.7%)	618 (39.5%)	5494 (38.1%)	0.052
Antenatal steroid exposure, *n* (%)	343 (82.9%)	1465 (82.5%)	4482 (84.2%)	1350 (86.3%)	12,096 (83.9%)	0.049
Cesarean delivery, *n* (%)	304 (73.4%)	1430 (80.6%)	4248 (79.8%)	1248 (79.7%)	11,860 (82.2%)	<0.001
1 min Apgar score	4 [2, 6]	5 [3, 6]	4 [3, 5]	5 [3, 6]	5 [4, 7]	<0.001
5 min Apgar score	7 [5, 8]	7 [6, 8]	7 [5, 8]	7 [6, 8]	8 [7, 9]	<0.001
Resuscitation severity score	2 [2, 2]	2 [2, 2]	2 [2, 2]	2 [2, 2]	2 [0, 2]	<0.001
Respiratory distress syndrome, *n* (%)	389 (94.0%)	1643 (92.6%)	4955 (93.1%)	1444 (92.3%)	9291 (64.4%)	<0.001
Surfactant treatment, *n* (%)	394 (95.2%)	1651 (93.0%)	4984 (93.6%)	1444 (92.3%)	9098 (63.1%)	<0.001
Hypotension, *n* (%)	145 (35.0%)	492 (27.7%)	2369 (44.5%)	656 (41.9%)	2161 (15.0%)	<0.001

Values are median [interquartile range] for continuous variables and n (%) for categorical variables. *p* values from Kruskal–Wallis test (continuous) or Pearson chi-square test (categorical). PDA = patent ductus arteriosus; VLBW = very low birth weight.

**Table 2:** Clinical outcomes by recorded PDA treatment strategy.

PDA Treatment Strategy	*N*	Pulmonary Hemorrhage, n (%)	In-Hospital Mortality, n (%)	Length of Stay, Days	PDA Drug Start, DOL
Prophylactic	414	41 (9.90%)	94 (22.71%)	70 [45, 94]	1.0 [0.0, 1.0]
Pre-symptomatic	1775	79 (4.45%)	192 (10.82%)	75 [56, 101]	2.0 [1.0, 4.0]
Symptomatic treated	5323	534 (10.03%)	576 (10.82%)	83 [59, 111]	5.0 [3.0, 10.0]
Symptomatic untreated	1565	192 (12.27%)	500 (31.95%)	68 [26, 109]	—
Asymptomatic untreated	14,423	333 (2.31%)	982 (6.81%)	51 [37, 69]	—

Values are n (%) for binary outcomes and median [interquartile range] for continuous outcomes. Pulmonary hemorrhage defined as KNN variable mph = 2 (confirmed pulmonary hemorrhage). In-hospital mortality defined as discharge status = death (dcd = 3). DOL = day of life).

**Table 3:** Pairwise inverse probability-weighted comparisons: pulmonary hemorrhage outcome.

Comparison	*N*	PH Rate, Exposed vs. Reference	IPW OR (95% CI)	*p* Value	ESS	Max |SMD| Post-IPW
**Pre-symptomatic vs. symptomatic treated**	**7049**	**4.48% vs. 10.05%**	**0.57 (0.46–0.72)**	**<0.001**	**6778.6**	**0.019**
Pre-symptomatic vs. symptomatic untreated	3320	4.48% vs. 12.33%	0.51 (0.39–0.66)	<0.001	3077.5	0.009
Prophylactic vs. pre-symptomatic	2175	9.95% vs. 4.48%	1.97 (1.31–2.96)	0.001	2078.7	0.04
Prophylactic vs. symptomatic treated	5698	9.95% vs. 10.05%	1.08 (0.78–1.51)	0.630	5658.0	0.055

Primary comparison (pre-symptomatic vs. symptomatic treated) shown in bold. Stabilized IPW with trimming at the 1st–99th percentiles of propensity score within each pairwise sample. PH rates shown are within the complete-case pairwise analysis sample and may differ marginally from the crude rates by full strategy group in Table 2. CI = confidence interval; ESS = effective sample size; IPW = inverse probability weighting; OR = odds ratio; SMD = standardized mean difference.

**Table 4:** Exploratory subgroup analysis: pre-symptomatic vs. symptomatic PDA treatment.

Subgroup	*N*	PH: Pre-Symptomatic vs. Symptomatic	Adjusted OR (95% CI)	*p* Value	*p* Interaction (per Domain)
GA < 28 w	3997	7.17% vs. 12.35%	0.61 (0.46–0.82)	<0.001	0.011
GA 28–31 w	2774	1.96% vs. 7.00%	0.34 (0.20–0.58)	<0.001	0.011
BW < 750 g	1603	11.27% vs. 16.27%	0.73 (0.50–1.06)	0.095	0.007
BW 750–999 g	2240	5.18% vs. 10.82%	0.50 (0.33–0.78)	0.002	0.007
BW 1000–1499 g	3119	1.48% vs. 6.12%	0.29 (0.16–0.52)	<0.001	0.007
Surfactant yes	6590	4.76% vs. 10.59%	0.51 (0.40–0.66)	<0.001	0.978
Hypotension yes	2836	10.43% vs. 16.02%	0.59 (0.43–0.80)	<0.001	0.169
Hypotension no	4213	2.20% vs. 5.27%	0.43 (0.28–0.65)	<0.001	0.169

Pre-symptomatic treatment versus symptomatic treatment. Consistent with the primary pairwise IPW comparison (Table 3). OR adjusted for gestational age, birth weight, sex, multiple birth, antenatal steroids, Cesarean delivery, respiratory distress syndrome, surfactant, and hypotension (stratifying variable excluded from the model within each stratum). *p* interaction tests binary effect modification within each domain using the full pre-symptomatic vs. symptomatic comparison set: GA < 28 weeks versus ≥ 28 weeks; BW < 1000 g versus ≥ 1000 g; surfactant use yes versus no; hypotension yes versus no. These do not represent omnibus tests across all displayed strata. CI = confidence interval; OR = odds ratio; PDA = patent ductus arteriosus.

## Data Availability

The data presented in this study are available on request from the corresponding author due to privacy and ethical restrictions governing the Korean Neonatal Network (KNN) registry data. Requests for access may be directed to the KNN Data Management Committee. Analysis code is available from the corresponding author upon reasonable request.

## Supplementary Materials

The following supporting information can be downloaded at https://www.mdpi.com/article/10.3390/children13080991/s1: Figure S1: Covariate balance before and after inverse probability weighting (IPW) for the primary comparison (pre-symptomatic vs. symptomatic PDA treatment); Figure S2: Propensity score distributions by treatment group for all pairwise comparisons; Figure S3: Pulmonary hemorrhage rates by PDA drug type; Figure S4: Postnatal day of first PDA drug administration by treatment strategy; Table S1: Early death rates by PDA treatment strategy; Table S2: Positivity sensitivity analyses using overlap (ATO) weights and PS-restricted IPW; Table S3: Sensitivity analyses excluding early deaths and including birth-year adjustment; Table S4: Baseline-only propensity score sensitivity analysis; Table S5: Pulmonary hemorrhage and mortality by PDA drug type; Table S6: Drug-type regression models for pulmonary hemorrhage.
