# Supplementary material for: PDA Treatment Strategy and Pulmonary Hemorrhage Risk in Very Low Birth Weight Infants: A Nationwide Cohort Study
Source: Children (Basel). 2026 Jul 26;13(8):991. doi: 10.3390/children13080991 (PMC13511359; doi:10.3390/children13080991)
Supplement: Supplementary file 1 [file children-13-00991-s001.zip › children-4426130-supplementary.pdf]

Supplementary Figures

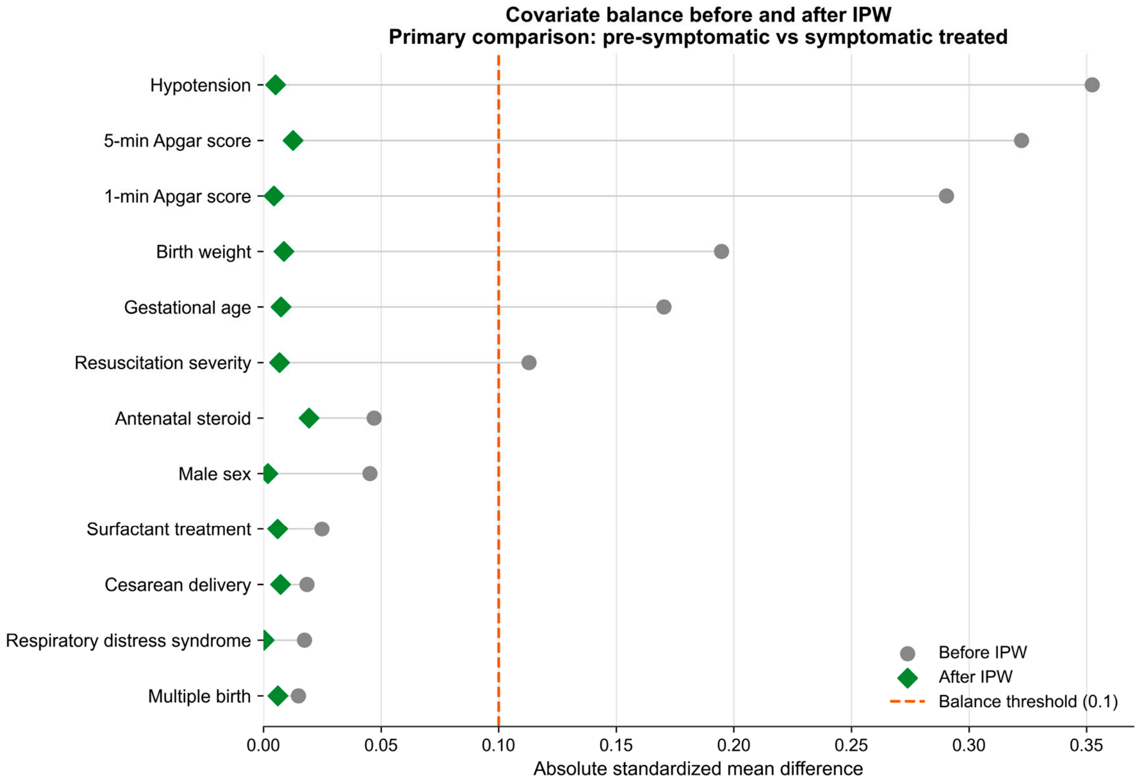

Figure S1. Covariate balance before and after inverse probability weighting (IPW) for the pri-mary comparison (pre-symptomatic vs. symptomatic PDA treatment). Absolute standardized mean differences (|SMD|) before (circles) and after (diamonds) inverse probability weighting for each covariate, shown for the primary comparison of pre-symptomatic versus symptomatic PDA treatment. The dashed vertical line marks the balance threshold (|SMD| = 0.10). IPW, inverse probability weighting; PDA, patent ductus arteriosus; SMD, standardized mean difference.

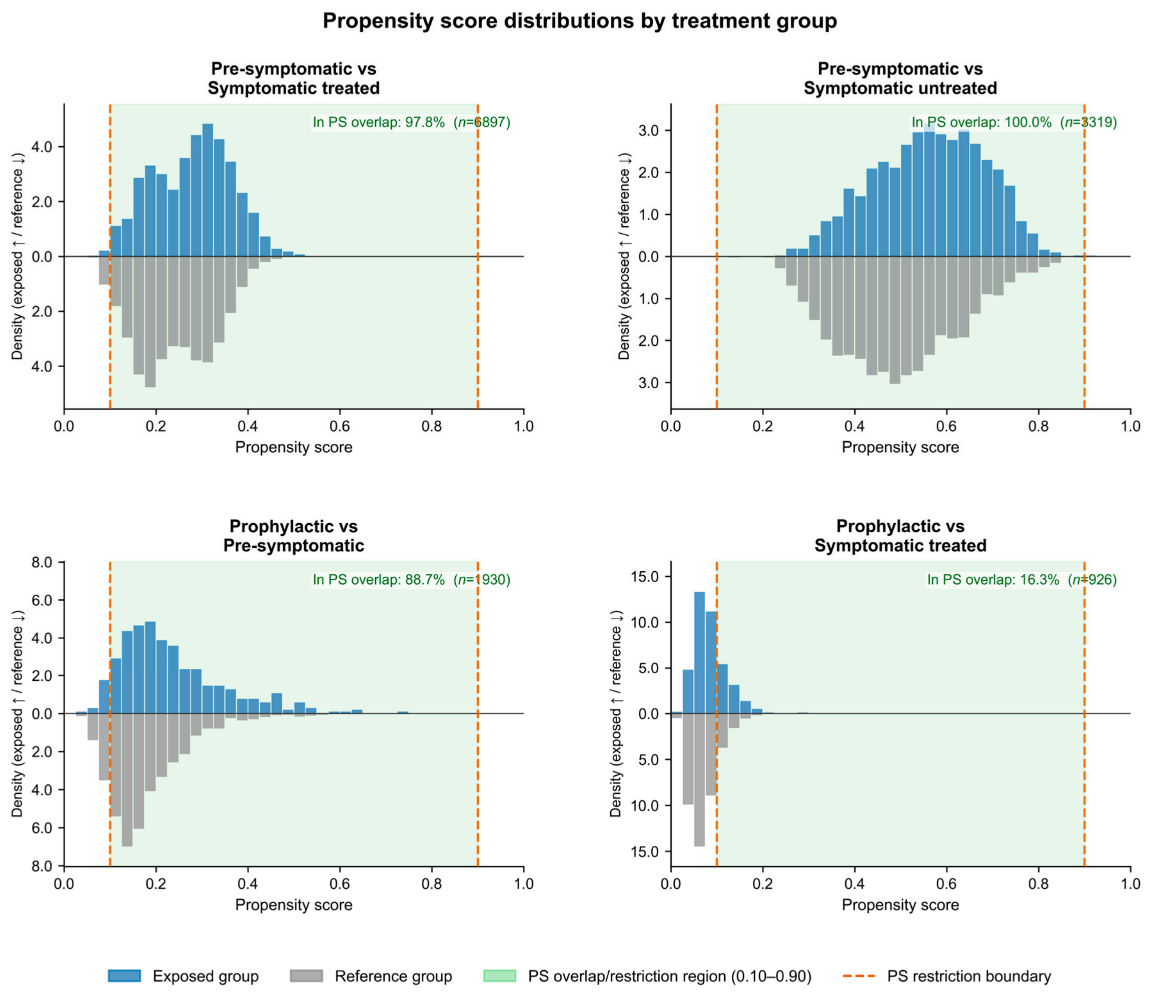

Figure S2. Propensity score distributions by treatment group for all pairwise comparisons. Mirror histograms showing propensity score (PS) distributions for the exposed group (bars above) and reference group (bars below, mirrored) for each pairwise comparison. Green shading marks the PS overlap/restriction region (0.10–0.90) used in PS-restricted sensitivity analyses; dashed lines indicate PS restriction boundaries. The prophylactic versus symptomatic treatment comparison showed severely limited overlap (16.3% within the PS overlap region), indicating substantial extrapolation in the primary IPW estimate for this comparison. The pre-symptomatic comparisons showed excellent overlap (97.8–100%). PDA, patent ductus arteriosus; PS, propensity score.

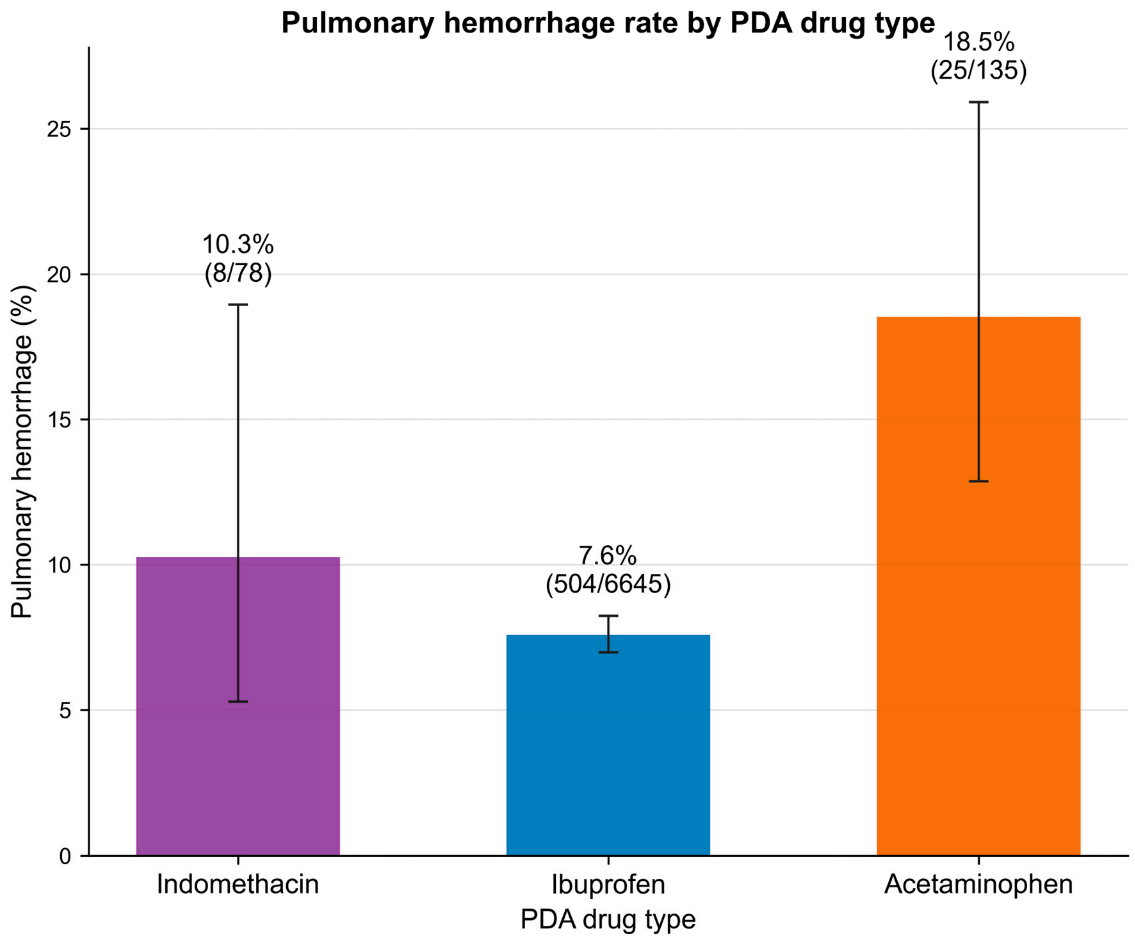

Figure S3. Pulmonary hemorrhage rates by PDA drug type. Observed pulmonary hemorrhage rates among pharmacologically treated infants by drug type (indomethacin, ibuprofen, or acetaminophen). These analyses are exploratory; non-ibuprofen groups were small and likely selected by clinical severity or contraindications. Error bars indicate Wilson 95% confidence intervals. PDA, patent ductus arteriosus; PH, pulmonary hemorrhage.

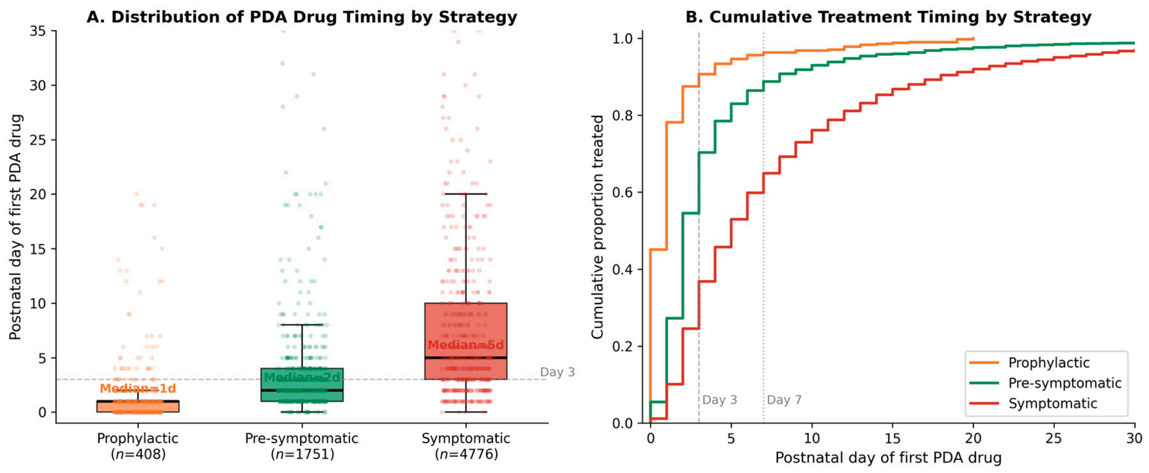

Figure S4. Postnatal day of first PDA drug administration by treatment strategy. (A) Box-and-strip plot of postnatal day of first PDA drug administration for prophylactic (n=408), pre-symptomatic (n=1751), and symptomatic (n=4776) treatment strategy groups. Boxes show interquartile ranges; horizontal lines indicate medians; whiskers extend to the 5th–95th percentile range; individual data points (random sample of 500 per group) are overlaid. The dashed horizontal line indicates postnatal day 3. (B) Cumulative proportion of infants receiving first PDA drug by postnatal day, by strategy group. Dashed and dotted vertical lines indicate day 3 and day 7, respectively. Median postnatal day of first drug differed significantly across groups (prophylactic: 1 day, IQR 0–1; pre-symptomatic: 2 days, IQR 1–4; symptomatic: 5 days, IQR 3–10; Kruskal-Wallis $p < 0.001$). Axes were truncated at postnatal day 35 (panel A) and postnatal day 30 (panel B) for readability; later outliers (to postnatal day 99 in the pre-symptomatic group and day 127 in the symptomatic group) are included in all summary statistics. PDA, patent ductus arteriosus.

Supplementary Tables

Table S1. Early death rates by PDA treatment strategy.

PDA strategy	*N*	Deaths by day 3, n (%)	Deaths by day 7, n (%)	Overall mortality, n (%)
Prophylactic	414	17 (4.11)	37 (8.94)	94 (22.71)
Pre-symptomatic	1775	13 (0.73)	34 (1.92)	192 (10.82)
Symptomatic treated	5323	27 (0.51)	99 (1.86)	576 (10.82)
Symptomatic untreated	1565	119 (7.60)	209 (13.35)	500 (31.95)
Asymptomatic untreated	14,423	382 (2.65)	537 (3.72)	982 (6.81)

Deaths by day 3 and day 7 are presented to characterize early mortality that may reflect treatment-opportunity bias in the symptomatic untreated group.

Overall mortality = in-hospital mortality (discharge status = death).

PDA = patent ductus arteriosus.

Table S2. Positivity sensitivity analyses using overlap (ATO) weights and PS-restricted IPW.

Comparison	*N* (primary)	Stabilized IPW (primary)	Overlap weights (ATO)	PS-restricted (0.10–0.90)	% in PS overlap region
**Pre-symptomatic vs symptomatic treated**	**7049**	**0.57 (0.46–0.72)**	**0.55 (0.40–0.76)**	**0.58 (0.46–0.72) [n=6,897]**	**97.8%**
Pre-symptomatic vs symptomatic untreated	3320	0.51 (0.39–0.66)	0.51 (0.35–0.74)	0.51 (0.39–0.66) [n=3,319]	100.0%
Prophylactic vs pre-symptomatic	2175	1.97 (1.31–2.96)	1.86 (1.00–3.46)	1.76 (1.16–2.67) [n=1,930]	88.7%
Prophylactic vs symptomatic treated	5698	1.08 (0.78–1.51)	1.10 (0.68–1.78)	0.97 (0.45–2.12) [n=926]	16.3%

Primary comparison (pre-symptomatic vs symptomatic treated) shown in bold.

Overlap (ATO) weights: each observation weighted by $P(A=0|X)$ for the exposed group and $P(A=1|X)$ for the reference group, down-weighting extreme propensity scores.

PS-restricted IPW: analytic sample limited to PS 0.10–0.90; stabilized weights recomputed within the restricted sample.

% in PS overlap region = percentage of infants within the 0.10–0.90 common support region.

ATO = average treatment effect in the overlap; CI = confidence interval; IPW = inverse probability weighting; OR = odds ratio; PS = propensity score.

Table S3. Sensitivity analyses excluding early deaths and including birth-year adjustment.

Sensitivity analysis	Comparison	Year adjusted	*N*	Weighted PH, exposed vs reference	Weighted risk difference, %	IPW OR (95% CI)	P value	Max abs SMD after IPW
Primary cohort	Pre–symptomatic vs symptomatic treated	No	7049	5.72% vs 9.56%	-3.84	0.57 (0.46–0.72)	<0.001	0.019
Primary cohort	Pre–symptomatic vs symptomatic treated	Yes	7049	5.72% vs 9.56%	-3.84	0.57 (0.46–0.72)	<0.001	0.019
Primary cohort	Pre–symptomatic vs symptomatic untreated	No	3320	5.56% vs 10.43%	-4.87	0.51 (0.39–0.66)	<0.001	0.009
Primary cohort	Pre–symptomatic vs symptomatic untreated	Yes	3320	5.56% vs 10.43%	-4.87	0.51 (0.39–0.66)	<0.001	0.009
Exclude deaths by day 3	Pre–symptomatic vs symptomatic treated	No	7010	5.35% vs 9.37%	-4.02	0.55 (0.43–0.69)	<0.001	0.02
Exclude deaths by day 3	Pre–symptomatic vs symptomatic treated	Yes	7010	5.35% vs 9.37%	-4.02	0.55 (0.43–0.69)	<0.001	0.02
Exclude deaths by day 3	Pre–symptomatic vs symptomatic untreated	No	3189	5.02% vs 8.84%	-3.82	0.55 (0.41–0.72)	<0.001	0.015
Exclude deaths by day 3	Pre–symptomatic vs symptomatic untreated	Yes	3189	5.02% vs 8.84%	-3.82	0.55 (0.41–0.72)	<0.001	0.015
Exclude deaths by day 7	Pre–symptomatic vs symptomatic treated	No	6917	5.07% vs 9.09%	-4.02	0.53 (0.42–0.68)	<0.001	0.02
Exclude deaths by day 7	Pre–symptomatic vs symptomatic treated	Yes	6917	5.07% vs 9.09%	-4.02	0.53 (0.42–0.68)	<0.001	0.02

Exclude deaths by day 7	Pre–symptomatic vs symptomatic untreated	No	3080	4.54% vs 7.51%	-2.97	0.59 (0.43–0.79)	<0.001	0.021
Exclude deaths by day 7	Pre–symptomatic vs symptomatic untreated	Yes	3080	4.54% vs 7.51%	-2.97	0.59 (0.43–0.79)	<0.001	0.021

Primary cohort: all infants with complete propensity score covariates.

"Exclude deaths by day 3/7" analyses remove infants who died before that threshold to address treatment-opportunity bias.

"Year adjusted" indicates birth year was added to the propensity score model.

CI = confidence interval; IPW = inverse probability weighting; OR = odds ratio; PH = pulmonary hemorrhage; SMD = standardized mean difference.

Table S4. Baseline-only propensity score sensitivity analysis.

Comparison	*N*	Weighted PH, exposed vs reference	Weighted risk difference, %	IPW OR (95% CI)	P value	Effective sample size	Max abs SMD after IPW
**Pre–symptomatic vs symptomatic treated**	**7049**	**5.12% vs 9.84%**	**-4.72**	**0.49 (0.39–0.62)**	**<0.001**	**6892.4**	**0.018**
Pre–symptomatic vs symptomatic untreated	3320	5.27% vs 11.13%	-5.87	0.44 (0.34–0.58)	<0.001	3139.0	0.01
Prophylactic vs pre–symptomatic	2175	9.07% vs 4.67%	4.4	2.04 (1.36–3.05)	<0.001	2082.8	0.041
Prophylactic vs symptomatic treated	5698	9.89% vs 10.03%	-0.15	0.98 (0.70–1.38)	0.924	5665.9	0.053

Baseline-only PS model excludes respiratory distress syndrome, surfactant treatment, and hypotension from the propensity score.

Primary comparison (pre-symptomatic vs symptomatic treated) shown in bold.

CI = confidence interval; IPW = inverse probability weighting; OR = odds ratio; PH = pulmonary hemorrhage; SMD = standardized mean difference.

Table S5. Pulmonary hemorrhage and mortality by PDA drug type.

Drug	*N*	Pulmonary hemorrhage	Mortality	Median GA, weeks	Median BW, g
Indomethacin	78	8 (10.26%)	12 (15.38%)	27.0	865
Ibuprofen	6,645	504 (7.58%)	733 (11.03%)	27.0	980
Acetaminophen	135	25 (18.52%)	16 (11.85%)	26.0	800

Ibuprofen was the predominant PDA drug (>90% of pharmacologically treated infants).

Indomethacin and acetaminophen groups were small and likely selected by clinical severity or contraindications to ibuprofen.

BW = birth weight; GA = gestational age; PDA = patent ductus arteriosus.

Table S6. Drug-type regression models for pulmonary hemorrhage.

Comparison	Model	OR (95% CI)	P value
Indomethacin vs Ibuprofen	Unadjusted	1.39 (0.67–2.91)	0.379
Indomethacin vs Ibuprofen	Covariate adjusted	1.47 (0.68–3.17)	0.332
Acetaminophen vs Ibuprofen	Unadjusted	2.77 (1.78–4.32)	<0.001
Acetaminophen vs Ibuprofen	Covariate adjusted	2.08 (1.30–3.32)	0.002

Ibuprofen is the reference category for all comparisons.

Covariate-adjusted model includes gestational age, birth weight, sex, antenatal steroids, surfactant, and hypotension.

Indomethacin vs acetaminophen comparison was not estimable (model singularity).

CI = confidence interval; OR = odds ratio; PDA = patent ductus arteriosus.
